# Evaluation of Nursing Students’ Experience of Clinical Placement in a Rural Setting Using CLES+T Scale

**DOI:** 10.3390/nursrep16040132

**Published:** 2026-04-13

**Authors:** Yangama Jokwiro, Qiumian Wang, Jennifer Bassett, Sandra Connor, Edward Zimbudzi

**Affiliations:** 1 Department of Rural Health Sciences, La Trobe University, Bendigo, VIC 3550, Australia; 2 Renal Service, Western at Home, Western Health, Melbourne, VIC 3021, Australia; lisa.wang@wh.org.au; 3 Department of Rural Health Sciences, La Trobe University, Shepperton, VIC 3630, Australia; j.bassett@latrobe.edu.au; 4 Department of Rural Health Sciences, La Trobe University, Mildura, VIC 3500, Australia; s.connor@latrobe.edu; 5 School of Nursing and Midwifery, Monash University, Clayton, VIC 3800, Australia; edward.zimbudzi@monash.edu

**Keywords:** clinical placement, clinical supervision, clinical learning environment, CLES+T, nursing student, student nurses, quality evaluation, rural health nursing workforce

## Abstract

**Background:** Nursing student experiences in the clinical learning environment have been described in many countries but less is known about student nurses in rural settings. **Aim:** To explore undergraduate nursing students’ experience of clinical placement in a rural setting and identify factors that influence their experience. **Methods:** A cross-sectional observational study was conducted with a convenience sample of 170 undergraduate nursing students in regional Victoria, Australia, who completed professional experience placements between January and June 2020. Following their placements, participants completed the Clinical Learning Environment, Supervision and Nurse Teacher (CLES+T) scale. Data were analysed using logistic regression models. **Results:** Completing clinical placements in medium to small rural towns or remote and very remote communities were associated with increased odds of high scores in the learning environment [odds ratio (OR) 2.90, 95% CI, 1.32 to 6.37; P = 0.01] and the supervisory relationship domains (OR 3.16, 95% CI, 1.40 to 7.14; P = 0.01). Female gender (OR 3.38, 95% CI, 1.12 to 10.19; P = 0.03), supervision by staff other than an educator (OR 2.71, 95% CI, 1.16 to 6.33; P = 0.02) and increased frequency of ad hoc (extra) supervision with a buddy nurse without the nurse educator (OR 2.55, 95% CI, 1.07 to 4.75; P = 0.03) were associated with increased odds of high scores in the role of nurse educator domain. **Conclusions:** In this study, nursing students reported valuing their exposure to smaller and more remote communities, the learning environments within rural and remote healthcare facilities, and the relationships they developed with supervising nurses. The findings also suggest that some students perceived greater value in supervision provided by clinical staff who were not in formal nurse educator or nurse facilitator roles. Given the limitations of the study, these observations should be interpreted cautiously and may warrant further investigation in broader contexts.

## 1. Introduction

Clinical placement is a core component of nursing education, providing students with opportunities to apply theoretical knowledge, develop clinical skills, and engage in reflective practice [1]. In Australia, a minimum of 800 placement hours is required for professional nursing registration [2]. Given the substantial time spent in these settings, student experience in the clinical learning environment (CLE), defined by Josiah (2018) in the Macy Foundation as “the social interactions, organisational cultures and structures, and physical and virtual spaces that surround and shape participants’ experiences, perceptions, and learning”, warrants attention [3]. Shaped by workplace values, norms, and culture, the CLE plays a critical role in fostering communication, critical thinking, and the development of professional identity [4,5,6]. However, nursing students often view CLE as a stressful part of their nursing education [7]. Student experience whilst on clinical placement has been reported to be influenced by multiple factors, including fear of failure, uncertainty in new clinical settings, isolation and intimidation as a result of a poor relationship with clinical educators and lack of practice opportunities [8,9,10].

In addition to the perceived challenges of the CLE, it is known that clinical placement experience is related to the perceived quality of education, learning outcomes, and the intention to join the nursing workforce after graduation [11,12,13]. Consequently, enhancing nursing students’ experience with the clinical learning environment has been a longstanding focus for nursing education providers, curriculum developers and nursing research. Although nursing students’ experience with CLE has been investigated in various contexts globally [8,12], to the authors’ knowledge, only one study was conducted in Australia, and none in Australian rural settings, areas that have higher reliance on nurses but face significant workforce shortages [13]. This notable gap highlighted the importance of assessing and improving nursing students’ experience with the CLE, given its correlation with workforce retainment [14,15].

To address this, the current study aims to explore undergraduate nursing students’ experience of clinical placement in a rural setting using the Clinical Learning Environment, Supervision and Nurse Teacher (CLES+T) scale [16,17]. It also seeks to identify key factors influencing these perceptions, with the goal of informing targeted strategies to enhance rural placement quality and workforce readiness.

The study is guided by the following research questions:

1. How do undergraduate nursing students perceive the quality of their clinical learning environment in rural Australian settings?
2. What are students’ experiences of supervision and nurse teacher support during rural clinical placements?
3. Which factors most significantly influence students’ experience of rural clinical placement quality?

## 2. Methods

### 2.1. Design

A cross-sectional design was used because of its ability to observe multiple outcomes and exposures in the study participants at one time point [18]. Cross-sectional studies can be considered particularly useful for studying the prevalence of a particular phenomenon, whether it is assumed to be the cause or the consequence, or both, in a defined population [18]. A cross-sectional design is best suited to explore undergraduate nursing students’ perceptions of clinical placement in the Australian rural setting and identify factors that influence these perceptions.

### 2.2. Demographic Data

Demographic data were collected to contextualise participants’ clinical placement experiences and explore potential influences on their perceptions. Variables included age, gender, course enrolment, and year of study, alongside geographic indicators such as remoteness and population size as measured by the Modified Monash Model [19,20,21]. In addition, the CLES+T instrument incorporated items examining the occurrence and frequency of supervision, supervisor qualifications, and the frequency of ad hoc (additional) supervision. These variables were utilised to investigate differences between and across participant groups.

### 2.3. Study Questionnaire

The CLES+T instrument is a questionnaire designed to measure students’ perceptions of the clinical learning environment [22]. It was chosen because among the eight instruments identified by Mansutti et al. (2017), in their systematic review of psychometric properties of instruments evaluating the CLE in nursing education, CLES+T was the most used, and has been validated in over ten different countries [23]. It consists of 34 items grouped into 3 domains, which include the learning environment, the supervisory relationship and the role of the nurse teacher, and seven subscales, which include pedagogical atmosphere, leadership style of the ward manager (WM), nursing care on the ward, the content of the supervisory relationship, the nurse teacher as enabling the integration of theory and practice, cooperation between placement staff and the nurse teacher and relationships between the student, mentor and nurse teacher [16]. Each item is rated on a Likert-type scale over 5 points (1 = fully disagree, 5 = fully agree, with higher score indicating more positive perception), allowing for a detailed evaluation of various aspects of the clinical learning experience. The mean score for each item and subtotal score for each subscale will be analysed. The scale is widely utilised to identify strengths and areas for improvement in clinical placements, inform educational interventions, and enhance the overall quality of nursing education and training. Some terminology from the CLES+T scale used in this study was modified to match terms used in the Australian nursing education system. These modifications were made to ensure the terminology was clear and easily understood by participants, given that the questionnaire was self-administered. The “pedagogical atmosphere” was renamed the “ward atmosphere”; the “ward manager” was changed to the “Nurse Unit Manager (NUM) (OR the immediate nurse leader)”; the “supervisor” was changed to “buddy nurse”; and the “nurse teacher” was changed to the “nurse educator/facilitator”. All changes were discussed and approved by the authors of the CLES+T scale (Saarikoski, M., Isoaho, H., Warne, T., & Leino-Kilpi, H) and the research team.

### 2.4. Study Sample

In 2020, all Latrobe University undergraduate students enrolled in nursing degree programs completing placements in non-metropolitan health centres in Victoria, Australia, were invited to take part in the study. The study focused on a cohort of students enrolled in a 3-year Bachelor of Nursing program (385) and a 2-year Enrolled Nurse Conversion or graduate entry program (47) designed for students with prior qualifications. Students were allocated to the rural facility randomly and did not have an opportunity to attend the facility prior to clinical placement for practice, although some facilities did provide an orientation session. Student hours at clinical placement varied depending on their year level and ranged from two to six weeks.

### 2.5. Setting

The Modified Monash Model (MMM) is an Australian geographical classification system that categorises locations from 1 (metropolitan) to 7 (very remote) based on their population size and remoteness [19,20,21]. In the context of this study, regional areas are defined as MM 2–3 (encompassing large to small regional centres), rural areas as MM 4–5 (including medium to small rural towns), and remote areas as MM 6–7 (covering remote and very remote communities) based on the MMM classification. Remoteness referred to the location of the clinical placement, not the student’s residential location prior to placement.

Hospitals located in areas classified as MM 2 (larger regional centres) typically have dedicated registered nurse educators or clinical nurse specialists (CNSs) assigned to individual wards, which are often organised by patient type (e.g., medical, surgical, day surgery). In contrast, hospitals situated in MM 3–7 locations (smaller rural and remote communities) tend to have mixed wards that accommodate both medical and surgical patients. These facilities are more likely to have urgent care centres rather than full emergency departments, and many also include aged care beds (typically 10–50), renal dialysis, medical imaging, community midwifery services, and various outpatient allied health services. Healthcare facilities located in MM 2 areas (regional centres) often discharge post-operative patients to smaller town hospitals in MM 3 or 4 locations as part of transition-to-home programs. Facilities in MM 2 areas generally manage higher-acuity patients compared to those in MM 3–5 locations. Some larger rural town health services (MM 3) are operationally linked to regional MM 2 hospitals and may have on-site nurse educators or receive weekly visits from nurse educators based at the MM 2 facilities. In some MM 3–5 settings, smaller hospitals operate under amalgamated management and governance structures, whereby multiple MM 4 or 5 facilities share a clinical educator who travels to support a student registered nurse (SRN) with assessment and debriefing.

These variations in facility management mean that student nurses in rural placements receive clinical supervision through a variety of models based on the accessibility of a nurse educator/facilitator. Supervision of SRNs in rural hospitals is allocated to the staff working the same shift, which may include a nurse practitioner, rural and isolated practice registered nurses (RIPRNs), or RN. SRN mentoring is not sequential, rather the SRN works with different staff of varying qualifications and capacity for each shift.

In MM 4 and 5 locations, hospitals have an alternate model of student mentoring ranging from a visiting nurse educator (1 nurse educator who works between 3 and 4 hospitals within a 100–200 km radius of each other) or a part-time nurse educator who ‘wears’ several hats at the same facility. Nurse educators in these hospitals have multiple roles that may include clinical educator, infection control, community nursing, associate nurse unit manager (ANUM) or RIPRN. Students are supervised by the available registered nurse, dependent on the skill mix rostered for that day or shift rather than being ‘buddied’ with the same RN for their complete placement.

### 2.6. Data Collection

The study questionnaire was uploaded to the Qualtrics Survey platform [24]. Upon finishing their clinical placement, students were approached to take part in the study by a clinical placement officer who is a university staff member not affiliated with the research. After each placement, an email invitation was sent, followed by one reminder to encourage students to complete the survey. Participants were provided with an information statement detailing the project and emphasising the voluntary nature of participation, with completion of the questionnaire indicating consent. The statement assured anonymity, as no personal identification was requested, and provided access to an anonymous questionnaire administered through Qualtrics.

### 2.7. Statistical Analysis

Distributions of demographic characteristics are presented as mean and standard deviation (SD) and categorical variables are reported as frequencies and percentages. The distribution of continuous variables was examined using histograms and Q–Q plots and statistically assessed using the Shapiro–Wilk test. First, chi-squared or *t*-tests (as appropriate) were used to examine differences in demographic characteristics in relation to learning environment, supervisory relationship and role of the nurse educator/facilitator scores. Second, internal consistency for each of the CLES+T domains was assessed using Cronbach’s alpha. In addition, inter-item correlations were reviewed within each domain to ensure item coherence and to verify that items measured the same underlying construct.

Multivariate logistic regression was performed to identify factors associated with lower mean scores for the learning environment, supervisory relationship and role of the nurse educator/facilitator. To analyse the three domains of the CLES+T scale using logistic regression, total composite scores were obtained from respective items associated with the domains. The total scores were then collapsed into two categories (low and high) using the median score as a cut off. All relevant independent variables regardless of their univariate results were included in the multivariate logistic regression models because they are important and warranted inclusion despite their statistical performance. Moreover, the study had few independent variables, which ensured that models were not overfitted. This approach reduced the likelihood of misleading R-squared values, regression coefficients, and *p*-values and improved the generalisability of the models beyond the current study. Potential factors included age, gender, course, year level, remoteness and population size as measured by the MMM, relationship with supervisor and frequency of supervision.

Due to the small number of participants in some MMM categories, data were grouped for logistic regression analysis to ensure meaningful comparisons and statistical reliability. Specifically, MMM classifications collapsed into two categories: MM 2–3 and above MM 3. This approach allowed for clearer interpretation of results while maintaining sufficient statistical power.

Statistical significance was indicated by a *p*-value of < 0.05 in all analyses. An initial assessment indicated that missing values did not follow a systematic pattern and accounted for 1.1% of the dataset so all questionnaires were included in the analysis. For the multivariable analysis, cases with missing values were excluded using listwise deletion. All analyses were performed with Stata version 11 [25].

### 2.8. Ethical Considerations

Ethics approval was granted by both the University’s Human Research Ethics Committee and the Human Research Ethics Committee of the healthcare agency involved (refence number, HREC19409). The explanatory statement provided to participants outlined the purpose of the study and the voluntary nature of participation. Additionally, no personally identifying data were required, and confidentiality was strictly maintained. Information on data storage of data and complaint procedures was also provided to participants [26].

## 3. Results

A total of 170 participants completed the survey, representing a 39% response rate from 432 individuals invited. Among respondents, 70% were aged 30 years or younger, and 150 (88%) were female. At least 72% were in the pre-registration 3-year program and over 50% of the participants were in the second year of their studies when they completed the survey. The majority of participants were supervised by an educator/facilitator during their last placement (75%) and only five participants came from metropolitan areas (MM 1) (Table 1).

Table 2 shows the mean and reliability scores of the seven domains of the CLES+T. The mean results for individual subscales ranged from 2.93 (1.10) for relationship among students, buddy nurse and nurse educator to 4.47 (0.84) for leadership style of the nurse unit manager or the immediate nurse leader. All domains showed good reliability (more than 80%).

The relationship between demographic characteristics of participants and the total learning environment, supervisory relationship and role of the nurse educator scores is shown in Table 3. There were no differences in the total learning environment scores with respect to age, course, year level, MMM, supervisor relationship and frequency of supervision (all *p* > 0.05). Participants from MM 4–7 had higher scores in the supervisory relationship domain compared to those from MM 3 and below (MD= −2.65, 95% CI −4.90 to −0.40; *p* = 0.02). In the role of nurse educator domain, female participants (MD= −5.31. 95% CI −8.44 to −2.18; 0.001) and those from MM 4–7 (MD= −2.47, 95% CI −4.72 to −0.21; *p* = 0.03) had higher scores compared to male participants and those from MM 3 and below, respectively.

**Figure 1:**
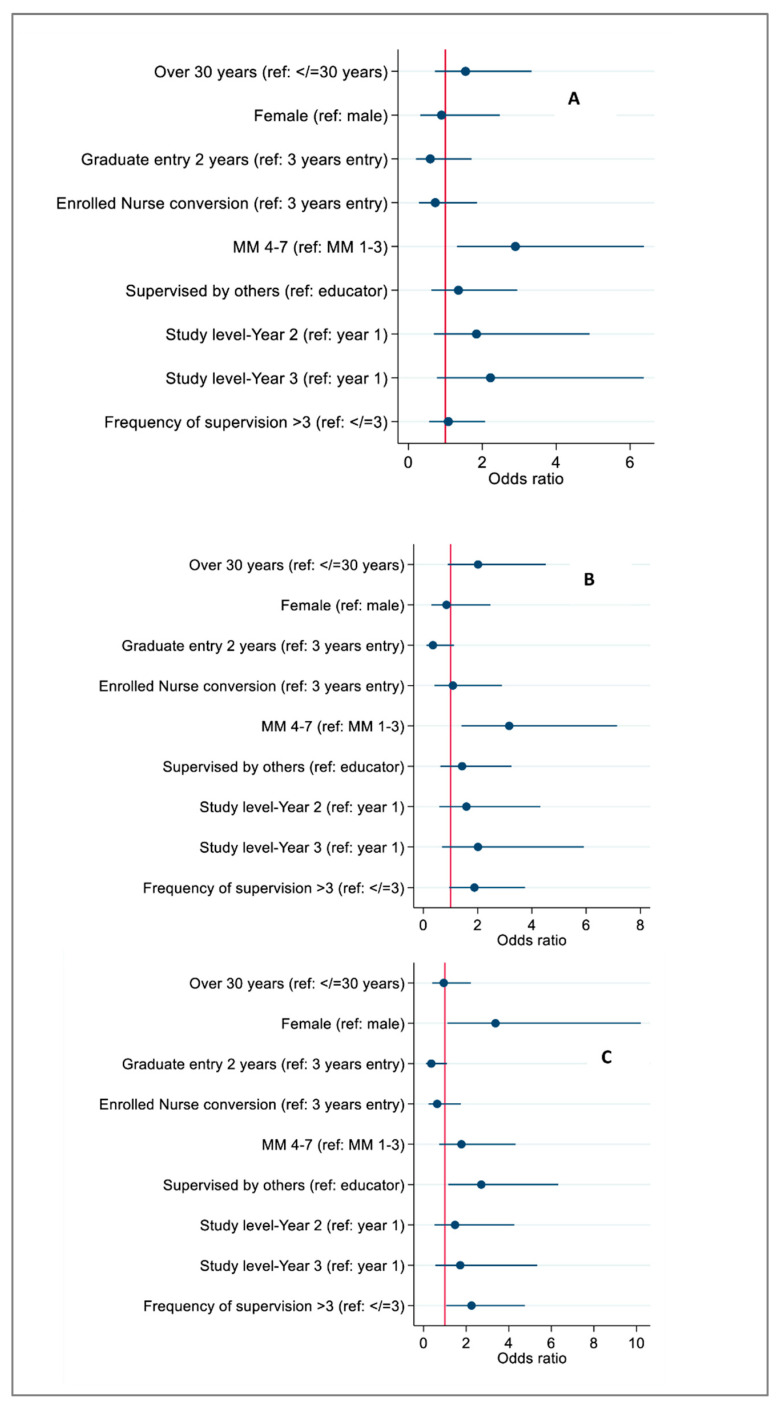
Association between demographic characteristics and learning environment (**A**), supervisory relationship (**B**) and role of the nurse educator scores (**C**).

Individuals from MM 4 and above compared to those from MM 3 and below had increased odds of scoring higher scores in the Learning Environment [odds ratio (OR) 2.90, 95% CI, 1.32 to 6.37; P = 0.01] and the supervisory relationship domains (OR 3.16, 95% CI 1.40 to 7.14; P = 0.01). Female gender (OR 3.38, 95% CI, 1.12 to 10.19; P = 0.03), supervision by staff other than an educator/facilitator (OR 2.71, 95% CI, 1.16 to 6.33; P= 0.02) and increased frequency of ad hoc (extra) supervision with buddy nurse without the nurse educator/facilitator (over three times) (OR 2.55, 95% CI, 1.07 to 4.75; P = 0.03) were associated with increased odds of high scores in the role of nurse educator/facilitator domain. Age, course and year level were not associated with any of the CLES+T domains (all *p* > 0.05) (Supplementary Table S1 and Figure 1A–C).

## 4. Discussion

This study aimed to explore undergraduate nursing students’ perceptions of clinical placement in the Australian rural setting. The findings suggest that the rural clinical learning environment can offer positive and supportive experiences for nursing students, particularly in relation to leadership and supervision. Students placed in more rural or remote settings tended to report more favourable perceptions of their learning environment, supervisory relationships, and support from nurse educators. Additionally, factors such as gender and the type and frequency of supervision appeared to shape how students experienced the role of the nurse educator, highlighting the complexity of clinical education in diverse rural contexts.

In this study, undergraduate students scored low in the role of nurse educator/facilitator domain. These findings contrast with previous studies among undergraduate students, which reported higher scores in the role of nurse educator/facilitator domain [27,28]. There are several reasons to explain this. First, nurse educators/facilitators are not always accessible to undergraduate students at regional hospitals due to high nurse educator–student ratios. Second, most nurse educators/facilitators in regional hospitals have multiple roles and this may lead to inconsistency in supervision [29]. While some students’ value exposure to a variety of facilitators on placement, the challenges associated with this approach include lack of confidence in supervising students by some nursing staff [30] and no official training in supervision of students [31].

Interestingly, our data suggest that the supportive nature of clinical placements in smaller and more remote communities may contribute to more positive perceptions of the learning environment and supervisory relationships. A possible explanation for this is that in remote and smaller hospitals, students are more likely to receive education, which aligns with their local cultural and academic needs [32]. To support this, a previous study reported that students on rural clinical placements perceived that rural health service staff were welcoming and supportive of their learning experiences [30] and they strived to know students not only as professionals, but the nature of their communication included getting to know them personally [33].

Our findings suggest that students may perceive effective educational support even when supervision is provided by staff other than designated nurse educators. Possible reasons for this could be that the increased exposure to a variety of clinical professionals broadens the students’ clinical experience and enhances their engagement with learning [34]. Additionally, being supervised or working with more than one ‘educator’ may offer students broader opportunities for engagement and exposure to diverse perspectives. This in turn can contribute to bridging the theory to practice gap, thus increasing the students’ satisfaction with the supervisory experience [1,34]. Additionally, it is established that on average rural nurses are older than city-based nurses, and that rural nurses report higher job satisfaction than their city counterparts [35,36,37]. These factors may increase the confidence the supervising nurses have when working with and supporting nursing students, leading to an enhanced experience for the student.

### 4.1. Strengths and Limitations

Our findings should be interpreted in light of the limitations and strengths of our study. First, the cross-sectional design of the study limits the ability to assess changes in undergraduate nursing students’ perceptions over time or determine the directionality of associations, thereby restricting conclusions about causality. Second, data were collected during the first year of the COVID-19 pandemic, a period marked by significant disruption to healthcare services and clinical education. These extraordinary circumstances may have influenced participants’ experiences and perceptions of clinical placement, potentially affecting how they responded to the survey items and introducing contextual bias into the findings. Additionally, the COVID-19 pandemic altered the delivery of curriculum at universities, which may have impacted students’ preparedness for clinical placement and therefore influenced the outcomes of this study. Third, the sample may not fully represent the broader population, given a response rate of 39%. However, this rate aligns with those reported in similar studies involving tertiary student populations [38].

Whilst acknowledging these limitations, there are several strengths inherent in this paper. Participation of invited students at 39% is in line with other studies of this nature. Additionally, students were evenly distributed across different year levels at university and levels of clinical experience.

### 4.2. Implications for Clinical Practice

The findings suggest that the placement of nursing students in smaller, remote communities may have the potential to positively influence their clinical education experiences. Students highly valued the immersive experiences and the strong relationships formed with supervising nurses in these settings. Notably, students tended to seek guidance from staff outside formal educator roles, which may suggest the potential value of informal mentorship by local clinical staff. This also implies that healthcare facilities should prioritise rural placements and provide mentorship training for all nursing staff, which recognises their pivotal role in student development. This approach has the potential to enhance student learning which may also contribute to building a sustainable nursing workforce in underserved areas.

## 5. Conclusions

This study contributes Australian-based evidence to a field largely informed by international research by exploring undergraduate nursing students’ perceptions of rural clinical placement. Students valued the opportunity to work in smaller and more remote communities, the supportive learning environments within rural and remote facilities, and the strong relationships formed with supervising nurses. Notably, they expressed a preference for supervision by staff who were not in formal nurse educator or facilitator roles—a finding that contrasts with previous studies and highlights the unique demands and multifaceted responsibilities of registered nurses in rural Australian settings. These insights underscore the need to consider local workforce structures and contextual factors when designing and supporting rural clinical placement experiences.

## Figures and Tables

**Table 1:** Demographic characteristics (n = 170).

Demographic Variables	Number (%)
Age (years)	
10–20	43 (25.29)
21–30	75 (44.12)
31–40	33 (19.41)
41–50	16 (9.41)
Over 50	3 (1.76)
Gender	
Male	19 (11.18)
Female	150 (88.24)
Prefer not to say	1 (0.59)
Course	
Pre-registration 3-year program	123 (72.35)
Graduate entry 2-year program	19 (11.18)
Enrolled Nurse Conversion	28 (16.47)
Year level on completion of last placement	
First year	24 (14.12)
Second year	87 (51.18)
Third year	59 (34.71)
Modified Monash classification of placement	
MM 1	5 (2.94)
MM 2	54 (31.76)
MM 3	71 (41.76)
MM 4	18 (10.59)
MM 5	20 (11.76)
MM 6	-
MM 7	2 (1.18)
Hospital status	
Public	148 (87.06)
Private	22 (12.94)
Qualification of the buddy nurse	
Personal Care Attendant/AIN	3 (1.78)
Enrolled Nurse	9 (5.33)
Registered Nurse	2 (2.37)
Educator/Facilitator	127 (75.15)
Associate Nurse Manager	2 (1.18)
Nurse Manager	24 (14.2)
Occurrence of supervision	
Did not have supervisor at all	3 (1.78)
A personal supervisor was named, but the relationship did not work	9 (5.33)
Named supervisor changed during the placement	4 (2.37)
Supervisor varied according to shift or place of work	127 (75.15)
Same supervisor had several students and was a group supervisor	2 (1.18)
Personal supervisor was named and our relationship worked	24 (14.20)
Frequency of ad hoc (extra) supervision	
Not at all	34 (20.00)
Once or twice during the course	44 (25.88)
Less than once a week	12 (7.06)
About once a week	29 (17.06)
More often	51 (30.00)

MMM—Modified Monash classification of placement; AIN—Assistant in Nursing.

**Table 2:** Mean and reliability scores of the seven domains of CLES+T scored by nursing students (n = 170).

Items	Mean (SD)	Inter-item Correlation	Alpha
The learning environment			
Pedagogical (ward) atmosphere			
Item 1 The staff members were easy to approach	4.32 (0.93)		
Item 2 I felt comfortable going to the ward at the start of my shift	4.26 (1.01)		
Item 3 During staff meetings (e.g., before shifts) I felt comfortable taking part in the discussions	3.77 (1.10)		
Item 4 There was a positive atmosphere on the ward	4.18 (1.02)		
Item 5 The staff members were generally interested in student supervision	3.99 (1.13)		
Item 6 The staff learned to know the student by their personal names	3.99 (1.13)		
Item 7 There were sufficient meaningful learning situations on the ward	4.29 (0.95)		
Item 8 The learning situations were multi-dimensional in terms of content	4.17 (1.03)		
Item 9 The ward can be regarded as a good learning environment	4.39 (0.94)		
Overall mean (SD)	4.39 (0.94)	0.55	0.92
Leadership style of the nurse unit manager or immediate nurse leader			
Item 1 The nurse unit manager (NUM) regarded the staff on her/his ward as a key resource	4.38 (0.85)		
Item 2 The NUM was a team member	4.39 (0.86)		
Item 3 Feedback from the NUM could easily be considered as a learning situation	4.00 (1.07)		
Item 4 The effort of individual employees was appreciated	4.47 (0.84)		
Overall mean (SD)	4.47 (0.84)	0.60	0.86
Nursing care on the ward			
Item 1 The ward’s nursing philosophy was clearly defined	4.14 (0.93)		
Item 2 Patients received individual nursing care	4.60 (0.67)		
Item 3 There were no problems in the information flow related to patients’ care	4.25 (0.94)		
Item 4 Documentation of nursing (e.g., nursing plans, daily recording of nursing procedures, etc.) was clear	4.45 (0.87)		
Overall mean (SD)	4.45 (0.87)	0.50	0.80
The supervisory relationship			
The quality of supervisory relationship			
Item 1 My supervisor showed a positive attitude towards supervision	4.29 (0.92)		
Item 2 I felt that I received individual supervision	4.35 (0.85		
Item 3 I continuously received feedback from my supervisor	4.24 (1.01)		
Item 4 Overall, I am satisfied with the supervision I received	4.34 (1.00)		
Item 5 The supervision was based on a relationship of equality and promoted my learning	4.31 (0.92)		
Item 6 There was a mutual interaction in the supervisory relationship	4.44 (0.77)		
Item 7 Mutual respect and approval prevailed in the supervisory relationship	4.36 (0.92)		
Item 8 The supervisory relationship was characterized by a sense of trust	4.38 (0.85)		
Overall mean (SD)	4.33 (0.79)	0.73	0.96
**Role of the nurse educator/facilitator**			
Nurse educator as enabling the integration of theory and practice			
Item 1 In my opinion, the nurse educator/facilitator was capable to integrate theoretical knowledge and everyday practice of nursing	4.47 (0.89)		
Item 2 The nurse educator/facilitator was capable of operationalising the learning goals of this clinical placement	4.43 (0.94)		
Item 3 The nurse educator/facilitator helped me to reduce the theory–practice gap	4.40 (0.95)		
Overall mean (SD)	4.40 (0.95)	0.83	0.93
Cooperation between buddy nurse/clinical staff and nurse educator/facilitator			
Item 1 The nurse educator/facilitator was like a member of the nursing team	4.05 (1.19)		
Item 2 The nurse educator/facilitator was able to give his or her pedagogical expertise to the clinical team	4.14 (1.10)		
Item 3 The nurse teacher and the clinical team worked together in supporting my learning	4.17 (1.07)		
Overall mean (SD)	4.17 (1.07)	0.81	0.93
Relationship among student, buddy nurse and nurse educator			
Item 1 The common meetings between myself, buddy nurse and nurse educator were comfortable experience	3.01 (1.55)		
Item 2 In our common meetings I felt that we are colleagues	2.81 (1.42)		
Item 3 Focus in the meetings was on my learning needs	2.93 (1.45)		
Overall mean (SD)	2.93 (1.10)	0.87	0.95

**Table 3:** Association between demographic characteristics of participants and the total learning environment, supervisory relationship and role of the nurse educator scores.

Variable	Demographic Characteristics	Learning Environment		Supervisory Relationship		Role of the Nurse Educator/Facilitator	
		Mean (SD)	*p*-Value	Mean (SD)	*p*-Value	Mean (SD)	*p*-Value
Age	Less than 30 years	71.84 (12.0)		34.37 (6.21)		30.34 (6.01)	
	Over 30 years	73.90 (9.82)	0.28	35.25 (6.65)	0.41	29.14 (7.12)	0.27
Gender							
	Male	71.05 (16.0)		34.56 (6.05)		25.24 (8.62)	
	Female	72.61 (10.72)	0.58	34.63 (6.42)	0.96	30.54 (5.86)	0.001
Course							
	Pre-registration 3-year program	72.63 (11.92)		34.97 (6.12)		30.39 (6.36)	
	Graduate entry 2-year program	71.47 (10.06)		32.11 (7.36)		29.28 (6.35)	
	Enrolled Nurse Conversion	72.52 (9.96)	0.92	34.89 (6.47)	0.20	28.71 (6.40)	0.41
Year level							
	First year	72.54 (9.24)		34.17 (5.72)		28.58 (6.01)	
	Second year	72.14 (11.61)		34.8 (6.46)		30.34 (6.74)	
	Third year	72.97 (11.97)	0.91	34.61 (6.52)	0.91	30.04 (5.94)	0.49
MMM							
	MM 1–3	71.55 (11.66)		34 (6.38)		29.38 (6.08)	
	MM 4–7	75.48 (9.98)	0.06	36.65 (5.86)	0.02	31.85 (6.92)	0.03
Qualification of the buddy nurse							
	Educator/facilitator	73.09 (10.52)		35.07 (5.84)		30.10 (6.13)	
	Others	70.67 (13.61)	0.23	33.34 (7.60)	0.13	29.63 (8.10)	0.69
Frequency of ad hoc supervision	Less or equal to 3	71.73 (12.22)		34.24 (6.13)		29.22 (6.36)	
	Over 3	73.31 (10.39)	0.37	35.10 (6.60)	0.38	30.86 (6.29)	0.10

MMM—Modified Monash classification of placement; SD—standard deviation; a—frequency of ad hoc (extra) supervision with buddy nurse (without the nurse educator/facilitator).

## Data Availability

The datasets used to support the conclusions of this article are included as tables in the results section. The data presented in this study are available on request from the corresponding author. The data are not publicly available due to privacy and ethical restrictions.

## Supplementary Materials

The following supporting information can be downloaded at: https://www.mdpi.com/article/10.3390/nursrep16040132/s1, Table S1: Evaluation of nursing students’ experience of clinical placement in the rural setting using CLES+T scale.

## Public Involvement Statement

No public involvement in any aspect of this research.

## Guidelines and Standards Statement

This manuscript was drafted against the Strengthening the Reporting of Observational Studies in Epidemiology (STROBE) guidelines for Epidemiological research.

## Abbreviations

ANUM	Associate Nurse Unit Manager
CLE	Clinical Learning Environment
CLES+T	Clinical Learning Environment, Supervision and Nurse Teacher scale
CNS	Clinical Nurse Specialist
MMM	Modified Monash Model
NUM	Nurse Unit Manager
RIPRN	Rural and Isolated Practice Registered Nurses
RN	Registered Nurse
SD	Standard Deviation
SRN	Student Registered Nurse
