# Supplementary material for: Evaluation of Nursing Students’ Experience of Clinical Placement in a Rural Setting Using CLES+T Scale
Source: Nurs Rep. 2026 Apr 13;16(4):132. doi: 10.3390/nursrep16040132 (PMC13119122; doi:10.3390/nursrep16040132)
Supplement: Supplementary file 1 [file nursrep-16-00132-s001.zip › nursrep-4184803-supplementary.pdf]

Supplementary Table S1: Association between demographic characteristics and learning environment, supervisory relationship and role of the nurse educator scores

**Variable**	**Learning environment**		**Supervisory relationship**		**Role of nurse Educator**	
	OR (95% CI)	p-value	OR (95% CI)	p-value	OR (95% CI)	p-value
Age (ref: less than 30 years)	-	-	-	-	-	-
Over 30 years	1.55 (0.72 to 3.33)	0.27	2.01 (0.90 to 4.51)	0.09	0.95 (0.40 to 2.22)	0.90
Gender (ref: male)	-	-	-	-	-	-
Female	0.90 (0.32 to 2.47)	0.83	0.85 (0.29 to 2.47)	0.77	3.38 (1.12 to 10.19)	**0.03**
Course (ref: Preregistration 3-year program)	-	-	-	-	-	-
Graduate entry 2-year program	0.59 (0.21 to 1.71)	0.33	0.35 (0.11 to 1.13)	0.09	0.36 (0.12 to 1.09)	0.07
Enrolled Nurse Conversion	0.73 (0.29 to 1.85)	0.51	1.09 (0.41 to 2.90)	0.87	0.64 (0.23 to 1.75)	0.38
Year level (ref: First year)	-	-	-	-	-	-
Second year	1.84 (0.69 to 4.90)	0.22	1.59 (0.58 to 4.30)	0.37	1.48 (0.51 to 4.26)	0.47
Third year	2.22 (0.78 to 6.36)	0.14	2.01 (0.69 to 5.91)	0.20	1.72 (0.55 to 5.34)	0.35
MMM (ref: MMM 1-3)	-	-	-	-	-	-
MMM 4-7	2.90 (1.32 to 6.37)	**0.01**	3.16 (1.40 to 7.14)	**0.01**	1.78 (0.73 to 4.32)	0.21
Qualification of the buddy nurse (ref: educator)	-	-	-	-	-	-
Others	1.35 (0.63 to 2.95)	0.44	1.43 (0.63 to 3.25)	0.40	2.71 (1.16 to 6.33)	**0.02**
*Frequency of ad hoc supervision (ref: 3 and below)	-	-	-	-	-	-
Over 3	1.08 (0.56 to 2.08)	0.82	1.88 (0.95 to 3.75)	0.07	2.25 (1.07 to 4.75)	**0.03**

MMM-Modified Monash Classification of Placement; OR-Odds ratio; CI-Confidence interval; *Frequency of ad hoc (extra) supervision with buddy nurse (without the Nurse Educator/Facilitator
